# Author Index for Volume 88

**DOI:** 10.1038/sj.bjc.6601553

**Published:** 2003-12-09

Abbruzzese, A 1971

Abdalla, S 1424

Abels, C 1462

Ablett, S 1661

Abrahamsson, J 1747

Abrams, KR 1191

Accordini, S 1666

Achard, J-L 1339

Açil, Y 1105

Acquaviva, AM 803

Adam, PJ 579

Adami, H-O 92, 1044

Adler, G 699

Aglietta, M 396

Ahlman, H 1747

Airoldi, I 1527

Akagi, M 796

Akaike, T 902

Akehurst, RL 31

Albertoni, C 996

Ali, S 1071

Ali-Osman, F 58

Allen, BJ 944

Allen, M 354, 1017, 1510

Allman, R 1649

Alonso, ME 117

Alquati, P 401

Althuis, MD 50

Amadori, D 320

Amat, S 1339

Ambagtsheer, G 314

Amberger, A 574

Anastasi, AM 996

Andersen, J 110

Andersen, K 1995

Anderson, NG 567

Anderson, R 1641

Ando, N 740

Andreasen, PA 110

Aneiros, JF 1702

Anglin, IE 1615

Ann Jordan, M 1794

Ansell, W 1516

Aoki, S 726

Appleby, P 1713

Appleyard, MVCL 1281

Arai, G 740

Arbyn, M 560

Ardern-Jones, A 487

Argraves, WS 871

Ariyoshi, Y 335

Arizono, Y 298

Arjona, D 109

Arnold, H 463

Arnold, J 270

Arnould, L 1587

Ashino, H 307

Ashton, SE 1592

Asosingh, K 855

Atzpodien, J 348

Augustsson, K 84, 1044

Austin, M 1851

Aytac, U 455

Baars, JW 1831

Baas, P 283

Babilas, P 1462

Babin, A 1925

Bachelot, T 181, 1721

Badve, S 1400

Baguley, BC 1844, 1942

Bai, AHC 1560

Baker, L 510

Balsari, A 1032

Bamberg, M 828

Bangur, C 887

Bannai, S 951

Banno, Y 606

Barbieri, V 1285

Barker, S 1641

Barnes, DM 487

Barnett, GH 1889

Baron, JA 1687

Baronchelli, C 401

Barrett, JH 1920

Barthel, B 1217

Bartlett, JMS 1263

Bartnik, W 1143

Barzanti, F 320

Bassal, S 1251

Batchelor, D 175

Batlle, A 432

Baudin, E 1537

Bauernhofer, T 1301

Baum, A, S 1301

Baumann, H 1773

Baxevanis, CN 1292

Baxter, JN 1271

Beale, P 1128

Bearchell, MC 1135

Beaujard, AC 1839

Beauregard, DA 1135

Beggs, FD 1549

Belimezi, M 1292

Bell, RL 1445

Bell, SM 1578

Bellenger, K 1160, 1844

Bello, MJ 109

Bellocco, R 1358

Bellon, N 1537

Belousova, N 1411

Belyakov, OV 767

Bénard, J 1741

Benoit, M 1839

Benoy, I 718

Beppu, T 902

Beral, V 1713

Berclaz, L 1781

Bergman, AM 1963

Berkson, RG 636

Bernsen, MR 424

Berrington de Gonzalez, A 1713

Berrino, F 1032

Berthois, Y 438

Bertino, JR 937

Bertoli, G 401

Besson, D 1669

Betts, J 90

Bex, A 1346

Bezdetnaya, L 154

Bhambra, U 1598

Bianchino, G 1956

Biasin, E 396

Bibby, MC 470

Bièche, I 863

Birch, JM 373

Birch-Machin, MA 98

Bird, K 257

Biron, P 181

Birringer, M 1948

Bishop, DT 1920

Bishop, JAN 1920

Bishop, L 354

Black, DM 1263

Blakey, DC 1592

Blay, J-Y 1721

Blay, JY 181

Blomqvist, P 1358

Blot, E 1207

Blot, WJ 684, 1687

Blotta, S 1285

Bodis, S 1786

Body, G 1339

Boehmer, G 1570

Boels, LA 560

Bogers, JJ 560

Boice, JD 382

Bökönyi, G 132

Boland, CR 1285, 1971

Bombardelli, E 965

Bommer, M 699

Bonfrer, JMG 1095

Boniver, J 502, 2004

Boogaerts, M 988

Boogerd, W 175

Borg, C 181

Bosetti, C 167

Bosma, AJ 1091

Bosq, J 1741

Bougnoux, P 1339, 1669

Boulay, J-L 420

Bourgeois, H 1669

Bourrié, B 438

Bousarghin, L 2002

Boutard, P 1925

Bovey, R 788

Boyle, P 58

Brach del Prever, A 396

Bradley, C 1160

Bradshaw, TD 470

Brady, G 567

Brborowicz, J 848

Brenner, H 1693

Bresaola, E 537

Breuning, MH 1675

Brewster, DH 74

Brickell, P 1641

Brindle, KM 1135

Brinton, LA 50

Broek, IV 855

Broekema, GJ 775

Brogan, DR 50

Broutet, N 1239

Brouwer, E 814

Brown, G 354, 1017

Brown, JM 666

Brueggen, J 1622

Brunner, M 782

Bruno, S 1527

Bucana, CD 796

Bufalino, R 1032

Buluwela, L 1071

Bundred, NJ 567

Buning-Kager, JCGM 1095

Burchill, SA 1191

Burk, RD 263

Burlage, FR 1012

Bussolati, G 702

Butcher, SK 748

Calabrese, P 491

Caliskan, E 1801

Calvert, NW 31

Camerini, A 1956

Campanile, N 996

Campbell, E 613

Camplejohn, RS 487

Cangiano, R 1956

Cannita, K 1507

Caporusso, L 491

Caraglia, M 1971

Carayon, P 438

Carey, DE 1411

Carminati, P 996

Carry, PY 1839

Casabonne, D 2002

Casalini, P 1032

Casas, A 432

Case, CP 548

Casellas, P 438

Cassani, G 996

Cassidy, J 624

Cassinelli, G 965

Cassoni, P 702

Catino, A 491

Catto, JWF 31

Cecchini, S 1175

Cemazar, M 1764

Cespon, C 940

Chamberlain, MP 1281

Chan, D 145, 1851

Chan, MWY 1560

Chao, M 1251

Chao, Y-Y 366

Chappell, SC 1310

Charalambous, MP 1598

Charnock-Jones, DS 237

Chatterjee, N 1584

Chau, I 354, 1017, 1510, 1859

Chauhan, SS 1327

Chauvin, F 181

Checkley, D 1592

Chelkowska, E 1361

Chen, C-D 1866

Chen, H-C 1058

Chen, TH-H 1866

Chen, W 1207

Chen, X 1878

Chenevix-Trench, G 270

Cheong, KF 413

Chernova, OB 1889

Chi, C-W 973

Chia, SK 1065

Chinol, M 996

Chinot, O 516

Cho, Y 1234

Choi, C-H 910

Chollet, P 1339

Chopin, D 388

Christen, RD 754

Chu, CM 210

Chua, SC 263

Chudecka-Głaz, A 848

Cianci, G 1507

Ciatto, S 1175

Ciolino, HP 599

Cittadini, A 1956

Clapisson, G 181

Clark, C 928

Clark, J 510

Classen, J 828

Clifford, GM 63

Clynes, M 1077

Coates, RJ 50

Coen, N 548

Cohen, LS 937

Coiffier, B 988

Colditz, GA 79, 1381

Coleman, N 257

Collette, L 843

Colowick, AB 1851

Colpaert, CG 718

Coluccia, C 702

Condom, E 878

Conradt, S 502, 2004

Cooke, TG 1263

Coombes, RC 1071

Cooper, CS 510

Cooper, PA 470

Cordes, N 1470

Corrias, MV 1527

Corso, S 396

Costantini, AS 55

Costanzo, F 1285

Counerotte, S 1111

Coursaget, P 2002

Couturier, J 1587

Cowdery, HE 137

Cowell, JK 1889

Cox, R 1939

Cramers, P 283

Cree, IA 1403

Crépin, M 1987

Creutzig, U 775

Croce, CM 1501

Croce, S 702

Crocetti, E 1175

Crosignani, P 47

Crucitta, E 491

Cui, R 1878

Cummings, J 1809

Cunningham, D 354, 1017, 1157, 1510, 1859

Curé, H 1339

Cutler, DL 1004

Czech, N 1105

Czene, K 1375

Dalen, H 161

Dalesio, O 283

D Alessandro, A 1971

D Alessio, V 996

Daling, JR 50

Damasceno, R 463

D Amico, C 491

Dang, NH 455

Dani, M 996

Däubler, P 1217

Dauplat, J 1339

Davico, L 47

Davidson, EJ 257

Davies, JM 1335

Davies, ML 202

Davies, P 1570

Daviet, L 579

De Bari, B 1956

De Boeck, G 314, 1979

de Boer-Dennert, M 814

de Boer, RF 648

de Bruijn, EA 314, 1979

de Bruin, M 957

de Campos, JM 109

de Carvalho Gomes, H 1570

de Gast, GC 175, 1346

de Jongh, FE 1199

De Lena, M 491

De Manzoni, G 537, 1666

De Santis, R 996

de Vries, EGE 1012

de Vries, MR 314

de Waal, M 1346

de Waal, MA 175

de Wit, R 843, 1199

Dębniak, J 848

Deckert, PM 937

Décorps, M 1439

Dedhar, S 1756

Dedicoat, M 1

Delap, LM 654

DeLellis, K 277

Delozier, T 1669

Delvaux, N 502, 2004

Denis, L 1004

Denkert, C 231

Denoux, Y 1587

Denoyelle, C 1207, 1631

Dent, J 1578

Depisch, D 1180

Depuydt, CE 560

Dervan, PA 871

Desai, M 42

Dette, S 217

Diamandis, EP 1101

Di Benedetto, M 1987

Dickinson, HO 1370

Dickman, PW 84

Dieckmann, K 496, 828

Diestra, JE 878

Dietel, M 231

Dijkstra, PU 1012

Dillner, J 1095

D'Incalci, M 965

Ding, H 1878

Dí Nícolantonío, F 1403

di Palma, A 803

Dippel, E 1157

Dirix, LY 718

Di Rocco, ZC 1507

Doak, SH 1271

Dogan, A 553

Doherty, VR 74

Donadio, M 702

Donato, F 401

Donnenberg, AD 223

Donnini, S 1484

Donovan, JL 1682

Double, JA 470

Douglas, JT 1411

Downes, CS 895

Downing, R 2002

Doyle, JJ 1185

Drake, MJ 822

Drew, PJ 4

Driouch, K 863

DuBois, RN 1445

Duc, A 1721

Duddy, PM 487

Duff, SE 1424

Duffaud, F 648

Duffy, JP 1549

Duffy, MJ 871

Dumontet, C 1794

Dunsche, A 1105

Durante, E 537, 1666

Durham, SE 90

Dworacki, G 1301

Easton, D 487

Ebert, MPA 1560

Eccles, DM 487

Edema, M 510

Edling, C 636

Edmunds, SC 1403

Edwards, A 1939

Edwards, JG 1553

Edwards, S 510

Edwards, T 1004

Eeles, RA 487

Egeland, T 36

Eggermont, AMM 314

Eguchi, K 808

Ehrnrooth, E 658

Eichler, HG 782

Ekbom, A 1687

El Kebir, FZ 1764

Ellis, LM 796

El-Magraby, MM 586

Elmberger, G 1229

Emerich, J 848

Emoto, M 237

Emri, S 167

Entz-Werle, N 1925

Errington, RJ 1310, 1649

Escolano, JM 940

Esposito, A 996

Etienne, A-M 502, 2004

Etterley, L 1128

Evans, BD 1844

Evans, SS 1773

Eyfjord, JE 933

EyTina, JH 1948

Fagioli, F 396

Falette, N 1794

Fallowfield, L 666

Fan, F 796

Fan, L 887

Faneyte, IF 406

Fang, B 918

Fang, J 902

Fanger, GR 887

Faraglia, B 1956

Farion, R 1439

Farmer, PB 470

Fazeny-Dörner, B 496

Feeney, GP 1310

Feldman, BM 1185

Fenton, BM 1453

Ferguson, J 1025

Feskanich, D 1381

Fétissof, F 1339

Fiche, M 1587

Ficorella, C 1507

Fielding, R 2006

Filippi, S 1484

Fischer-Colbrie, R 1747

Fisher, C 510

Fitzgerald, JJ 1549

Fitz-simons, N 1077

Flamini, E 320

Flanagan, J 270

Fletcher, GC 579

Flohr, P 510

Florl, AR 1932

Fodstad, O 1995

Foekens, JA 1084

Foidart, J-M 1111

Folkard, M 767

Folkes, L 1160

Fong, D 574

Fontana, A 47

Fontana, MG 401

Foo, W 2006

Forrest, S 42

Fosså, SD 36

Fox, SB 718

Fraggetta, F 537

Fraire, F 702

Franceschi, S 63, 167, 1388, 1713

Frapolli, R 965

Fraser, JA 1263

Frelinger, JA 1453

Frelinger, JG 1453

Friebe-Hoffmann, U 1301

Friedlos, F 1622

Friis, S 684, 1687

Fuchs, CS 79

Fujii, T 1213, 1883

Fujita, H 606

Fukuchi, M 1735

Fukuchi, T 1883

Fukuda, H 432

Fukuhara, M 521

Fukunaga, A 1234

Fukuoka, M 335

Fukushima, M 342, 957

Fumoleau, P 648

Furuse, K 342

Gabbert, HE 918, 1801

Gabrecht, T 1781

Gajalakshmi, V 1388

Galbraith, S 1160

Galeotti, T 1956

Galiègue, S 438

Gallagher, WM 871

Galmarini, CM 1794

Gambini, C 1527

Gamelin, E 1669

Gammon, MD 50

Ganesan, R 586

Garbrecht-Buettner, S 1570

García del Muro, X 878

Gardy, R 1424

Garinis, GA 206

Gaskell, M 470

Gastl, G 574

Gatter, K 553

Gatter, KC 1065

Gaub, M-P 1925

Gaut, R 1920

Gerber, SA 1453

Gerharz, CD 918, 1801

Gerhold, M 754

Germà-Lluch, JR 878

Germano, IM 1277

Germer, CT 115

Gerson, SL 1004

Gervasi, G 1175

Geyer, C 1004

Ghavamian, R 263

Ghilchik, MW 630

Gilchrist, R 487

Gilham, DE 1119

Gill, V 1659

Gillatt, D 1682

Gillis, C 1708

Gilly, FN 1839

Giordano, S 396

Giovannucci, E 79

Gippner-Steppert, C 1084

Girault, I 863

Glanzmann, C 1786

Glaspy, J 1851

Glehen, O 1839

Gnanapragasam, VJ 1432

Goel, A 1285, 1971

Goetz, AD 1004

Gogas, H 983

Going, JJ 1263

Gollnick, SO 1773

Goluda, M 848

Gonzalez-Gomez, P 109

Gonzalez-Porque, P 940

Gooderham, NJ 1598

Gooding, W 223

Goovaerts, G 718

Goto, K 808

Gottesman, MM 1327

Grabowski, P 115

Graff, BA 291

Granato, AM 320

Grant, K 163

Graveland, WJ 1199

Green, J 1713

Greenberg, ML 1185

Greene, LM 871

Gregor, M 217

Gregoriou, A 1168

Gress, TM 699

Griffiths, AP 1271

Griffiths, JR 1592

Grimer, R 510

Gritzapis, AD 1292

Grobbee, DE 1394

Groenewegen, G 1346

Gudmundsdottir, K 933

Guetens, G 314, 1979

Guibert, B 1839

Guida, M 491

Guillemin, F 146

Gulliford, MC 1025

Gumbrell, L 1160, 1844

Gunnarsdóttir, KA 832

Gunnell, D 1682

Gunsilius, E 574

Günther, W 463

Gupta, A 1400

Gustafsson, B 424

Habbema, JDF 843, 1054

Haboubi, N 1424

Hackshaw, AK 1047

Hählen, K 775

Hahn, EG 1248

Haider, K 1180

Haites, NE 928

Håkansson, A 424

Håkansson, L 424

Halbert, G 1160

Halbert, GW 1844

Hall, GD 1578

Halls, J 1939

Hamada, H 951

Hamada, K 1909

Hamada, Y 298

Hamdy, FC 39, 1432, 1682

Hammal, DM 1370

Hammond, LA 1004

Han, C 1065

Hancock, BW 1335

Handgretinger, R 1874

Hanke, B 1248

Hanley, G 1445

Hanover, JA 1327

Hansen, S 102

Haque, S 354

Harada, H 342

Harington, JS 1361

Harris, A 553

Harris, AL 579, 718, 1065

Harris, MA 654

Hart, AAM 707, 1091

Hart, SL 1641

Harvey, VJ 1844

Hasan, J 1828

Hasilik, A 733

Haslett, C 1809

Hastings, JM 237

Hatton, C 553

Hawkins, RE 1119

Hazato, T 307

He, J 1878

Hedberg, Y 1417

Hedderich, J 1105

Hedley, AJ 2006

Heiden, T 478

Heider, U 1406

Heikaus, S 1801

Heine, B 123, 1217

Helms, G 478

Hemminki, K 1375

Henderson, B 277

Henderson, BW 1773

Heney, D 1191

Henze, G 775

Herbst, H 733

Herrero, R 1388

Herrmann, R 420

Hertzog, PJ 145

Hervieu, V 1794

Heydon, RT 470

Hickish, T 1859

Hicklin, DJ 796

Hida, Y 1234

Higashiyama, S 327

Higgins, M 636

Hildenbrand, R 1157

Hill, A 1017

Hill, AD 871, 1077

Hill, M 1017, 1510, 1859

Hiller, L 586

Hiraoka, K 1234

Hirohashi, K 1894

Hirohashi, S 726

Ho, GYF 263

Hochhaus, A 1157

Hodgson, SV 487

Hoekman, K 957

Hoey, RP 567

Hoffmeister, B 1217

Hojo, F 808

Hole, D 1708

Holford, TR 58

Holland, CM 237

Holly, JMP 1682

Hölmich, LR 832

Holtkamp, MJ 1831

Holyoake, TL 983

Holz, B 1570

Holzmann, K 217

Hone, P 1939

Hong, L 1631

Hongyo, T 1584

Höpfner, M 115

Hopster, D 251

Horenblas, S 1095, 1346

Hori, H 307

Horváth, A 132

Hose, C 599

Hosoe, S 342

Hostanska, K 1786

Hough, RE 1335

Hovig, E 1995

Howe, FA 1592

Hsieh, CJ 217

Hsieh, J-L 1492

Hsieh, PS 210

Hsieh, SY 210

Hsing, AW 263

Hsu, C-Y 187

Hsu, T 1942

Hu, D-E 1135

Hu, Y 1605

Huang, C 1727

Huang, H-L 366

Huebner, K 1501

Huilgol, NG 1584

Hultdin, M 593

Hungerford, JL 1403

Hunter, DJ 1381

Hupp, TR 1281

Hurren, JS 1403

Hussain, SA 586

Iddon, J 567

Iftner, T 1570

Ihm, K 1874

Iino, Y 1909

Ikematsu, S 1522

Ikeya, T 1909

Imai, K 1543

Inada, M 1318

Inayama, S 307

Ingber, S 1185

Ingles, S 277

Inoue, Y 887

Ippolito, A 996

Irlam, J 1119

Irthum, B 516

Ishibashi, M 195

Ishida, E 1223

Ishikawa, M 1883

Isla, A 109

Ito, A 1318

Ito, H 18

Ito, T 18

Ito, Y 327

Itoh, T 1234

Iveson, T 1859

Iwahashi, M 530

Iwamoto, Y 342

Iwata, T 1883

Izquierdo, MA 878

Jack, RH 1025

Jacobs, IJ 251

Jakob, C 1406

Jakobs, C 447

Jakobsen, A-M 1747

James, ND 586

Jameson, MB 1844

Jamieson, SMF 1942

Jan, J-S 187

Janka-Schaub, GE 775

Janne, P 502, 2004

Janot, F 1741

Jansen, R 1346

Jaurand, MC 388

Jayson, GC 654, 1828

Jeȩdryka, M 848

Jenkins, GJS 1271

Jensen, A 362

Jensen, AB 658

Jiang, R-S 187

Jimenez-Puente, A 1702

Jochum, M 1084

Jodrell, D 1809, 1859

Johansen, C 1698

John, M 510

Johnsen, SP 684

Johnson, A 593

Johnson, P 1152

Jonasson, JG 933

Jones, DR 1191

Jones, JL 1553

Jordan, MA 1793

Jörgensen, HG 983

Judson, I 510, 1128

Juillerat-Jeanneret, L 788

Jung, K 1101

Jung, M 217

Jung, Y-K 910, 910

Jung, YD 796

Jungbluth, A 937

Kaaijk, P 775

Kaaks, R 1394

Kaas, R 707

Kadhim, MA 548

Kadomatsu, K 1522

Kageyama, Y 740

Kainz, C 988

Kakinuma, R 808

Kalifa, C 1925

Kamath, K 1794

Kameoka, S 1900

Kametani, M 1038

Kameyama, K 1727

Kaneko, K 18

Kanzawa, T 1277

Kao, H-W 1058

Karpińska, G 848

Kasamatsu, T 245

Kaspers, GJL 775

Katagiri, A 18

Katakami, N 335

Kato, H 1735

Kato, K 1234

Kato, T 1900

Katoh, H 606, 1234

Katsumata, N 245

Kawaguchi, M 796

Kawaguchi, N 327

Kawahara, M 342

Kawakami, S 740

Keizer, HJ 843

Kelleher, MT 354

Kelsell, DP 1403

Kémény, J-L 516

Kempen, I 1111

Kennedy, SM 1077

Kéri, G 132

Kersten, MJ 175, 1346

Kestell, P 1844

Key, T 1394

Khalil, T 516

Khan, OA 1549

Kheuang, L 388

Kidwai, N 1400

Kievit, J 1675

Kießlich, R 217

Kihara, K 740

Kikuchi, H 195

Kim, I-K 910

Kim, M 1411

Kimura, T 1900

Kingsmore, D 1708

Kingston, RE 839

Kinnon, C 1641

Kinoshita, M 1727

Kirova, Y 388

Kirwan, JM 839

Kitahara, T 11

Kitajima, M 726

Kitchener, HC 257

Klastersky, J 502, 2004

Kliesch, S 828

Klijn, JGM 1084

Klimek, M 848

Klingebiel, T 1874

Klump, B 217

Kneeshaw, PJ 4

Knoop, A 102

Knuutila, S 1914

Ko, Y-C 366

Kobayashi, M 1900

Kobayashi, S 1727

Kodama, T 808

Koga, F 740

Kogner, P 478

Kola, I 137

Kölby, L 1747

Kolonel, L 277

Komata, T 1277

Komuta, K 342

Kondo, S 1234, 1277

Kondo, T 951

Kondo, Y 1277

Konishi, K 18

Konishi, N 1223

Konstantopoulos, K 981

Korbelik, M 760

Kornek, GV 1180

Korse, CM 1095

Kosmas, C 1168

Kothari, MS 1071

Koyama, T 1909

Kraemer, M 1987

Krag, C 832

Krasnykh, V 1411

Kraszewska, E 848

Krejcy, K 167

Kretsovali, A 1292

Krieg, A 918, 1801

Krieg, T 918

Krishnan, KJ 90

Kristiansen, G 231, 1101

Kruyt, FA 957

Kruyt, FAE 447

Krysander, L 424

Ku, SL 413

Kubo, S 1894

Kubota, K 808

Kubushiro, K 1883

Kudoh, S 335

Kuehn, M 828

Kugu, K 1213

Kühnel, T 123

Kumagai, J 740

Kumar, S 1424

Kunitoh, H 808

Kupryjańczyk, J 848

Kurahashi, T 18

Kuriyama, T 25

Kuriyama-Matsumura, K 951

Kurokawa, T 1234

Kurosu, K 25

Kuss, I 223, 1301

Kuwano, H 1735

Kuzumaki, N 606

Kvinnsland, Y 291

Kwasny, W 1180

Kwiatkowski, F 516, 1339

Kyprianou, N 1615

Laccabue, D 965

Lacher, U 699

Laffer, U 420

Lai, J 270

Lai, M-D 1492

Laín, S 636

Lam, T-H 2006

Lambe, M 1358

Lambert, PC 1191

Lancashire, RJ 1035

Landberg, G 1417

Landini, A 1175

Landthaler, M 1462

Landuyt, B 1979

Landuyt, W 1979

Lane, DP 636

Lanfiuti Baldi, P 1507

Lang, F 1180

Lang, P 1874

Lange, N 1781

Langelotz, C 1406

Lækholm, A-V 102

lannucci, A 537

Lans, TE 314

Lanzi, C 965

La Pinta, M 401

Laskey, RA 257

Latorre, A 491

Lau, EMC 2006

La Vecchia, C 167

Lavender, T 839

Leboulleux, S 1537

Le Cesne, A 181

Lecomte, C 388

Lee, C-H 366, 1492

Lee, EJ 754

Lee, I-M 679

Lee, SN 210

Lee, Y-F 510

Leek, RD 1065

Lefebvre, JL 11

Legrain, P 579

Leigh, I 553

Leitzmann, MF 87

Lenoir, G 1741

Leong, C-O 470, 599

Leong, RWL 1560

Leong, T 1251

Leoni, B 996

Leroux, G 1741

Leroy, K 388

Leung, GM 2006

Leung, HY 822, 1432

Leung, W 1874

Leung, WK 1560

Levy, F 388

Li, C 1424

Li, J 1698

Li, M 263

Li, Y 944, 1878

Liang, XJ 1327

Liaw, SF 210

Liaw, YF 210

Libert, Y 502, 2004

Licence, D 237

Lidereau, R 863

Liebsch, G 1462

Liem, AA 1281

Lightfoot, T 1598

Lin, H-L 973

Lin, J-C 187

Lin, KH 210

Lin, L-M 366

Lin, W-C 1058

Linari, A 396

Linder, S 1229

Lindskog, M 478

Lindstedt, R 996

Linehan, R 1077

Liu, D 1727

Liu, J 1942

Liu, S-M 263

Liu, T-Y 973

Liu, W 796

Livni, N 1071

Ljungberg, B 1417

Lloyd, D 1939

Loader, JA 579

Loadman, P 1160

Loaiza-Pérez, AI 599

Locker, G 782

Lofts, F 1859

Loizidou, M 163

Lomas, J 117

Look, MP 1084

Loonen, AH 775

Lord, EM 1453

Lord, JM 748

Lorigan, PC 1335

Lortholary, A 1669

Lorusso, V 491

Lotz, V 1669

Loves, WJP 1963

Loy, V 828

Lualdi, S 1527

Luboinski, B 1741

Lucas, AR 104

Lucas, S 2002

Lüdicke, F 1781

Lui, W-Y 973

Lutz, P 1925

Lutzke, ML 1566

Lynge, E 362

Lyons, GR 1411

Ma, J 814

Maaser, K 1217

MacGrogan, G 1587

MacKinnon, AC 1809

MacNeil, A 1566

Madarame, J 327

Mader, RM 782

Madon, E 396

Mądry, R 848

Maeda, H 902

Magyezi, J 2002

Mahmud, N 895

Mahotka, C 918, 1801

Maier, P 1773

Maihöfner, C 1598

Maillard, K 510

Maissoneuve, P 537

Major, AL 1781

Makita, F 1909

Malæos, N 1168

Mlandsmo, GM 1995

Malfertheiner, P 1560

Maliepaard, M 814

Mallo, H 1346

Malone, KE 50

Mamalaki, A 1292

Mamot, C 420

Manegold, C 167

Manfredi, V 996

Mangham, DC 510

Manolis, EN 206

Mansfield, PF 796

Manson, WL 1012

Marais, R 1622

Marcellin, L 1925

Marec-Berard, P 1925

Margreiter, R 574

Markham, AF 1578

Markowska, J 848

Marosi, C 496

Marotta, A 1756

Marquez, N 1310

Marsh, C 1432

Martelli, ML 1285

Martin, EA 470

Martin, PM 438

Martínez-García, C 1702

Martus, P 1248

Masala, G 47

Masereel, B 1111

Mason, M 1152

Massey, A 1017

Masters, J 1823

Masuda, ES 910

Masuda, N 335, 1735

Masumoto, N 1883

Masuya, D 1727

Mathieu, MC 1587

Matrisian, LM 1445

Matsuda, K 530

Matsukura, S 521

Matsumoto, T 808

Matsuno, Y 245

Matsushita, S 1038

Matsuura, N 327

Matter, A 1622

Maycroft, K 1622

Maynard, M 1682

Mazzei, A 491

McCaffery, K 421

McCann, AH 871

McCarty, MF 796

McCrystal, MR 1844

McDermott, EW 871, 1077

McGlashan, ND 1361

McGlynn, AP 895

McIntyre, DJO 1592

McKay, M 1251

McKeage, MJ 1942

McKeague, AL 125

McKean-Cowdin, R 277

McKelvey-Martin, VJ 895

McKenna, HDB 74

McKenzie-Edwards, E 487

McKerr, G 895

McLaren, J 1553

McLaughlin, JK 684, 832, 1687

McLeod, HL 928

McLimont, M 1185

McMillan, L 748

McNally, R 373

McNeill, PD 887

McNulty, H 895

Medri, L 320

Mégraud, F 1239

Mehta, P 822

Meijer, CJLM 1388

Mekid, H 1764

Melcher, AA 1352

Mallemkjær, L 832, 1687

Melman, A 263

Ménard, S 1032

Menéndez, S 636

Menetrier-Caux, C 1721

Menounos, PG 206

Menton, M 1570

Menton, S 1570

Merckaert, I 502, 2004

Messori, L 1484

Metzger, U 420

Meyer, N 1925

Meza, LA 1851

Michael, BD 767

Michalski, A 1641

Michaud, DS 79

Mild, G 420

Miligi, L 47

Mills, GB 455

Miñarro-del, Moral, RM 1702

Mir, LM 1764

Mitamura, K 11

Miyamoto, M 1234

Miyamoto, Y 902

Miyaura, C 1318

Miyazaki, K 521

Miyazaki, T 1735

Miyazawa, J 1543

Mizunuma, H 237, 1543

Mizutani, Y 11

Molife, R 1335

Møller, H 1025

Møller, S 832

Moneghini, D 401

Monneuse, O 1839

Monnier, A 1669

Monsaert, C 120

Moormann, A 1566

Moran, JP 1453

Morazzoni, P 965

Morbidelli, L 1484

Mordenti, GL 320

Morelli, MF 1507

Morese, R 1507

Morgan, AB 31

Morgan, DW 1445

Morgan, WE 1549

Morikawa, T 1234

Morimoto, C 455

Morioka, T 342

Morishita, Y 1909

Moriya, T 25

Moriya, Y 726

Morlière, P 154

Morris, LS 257

Morrow, M 1400

Mothersill, C 767

Mothersill, CE 548

Moulton, GG 887

Mouncey, P 31

Mucci, LA 841

Mueller, F 699

Mueller, M 782

Mühr-Wilkenshoff, F 115

Mukai, M 1883

Mukai, T 521

Mullan, F 895

Mulsant, P 1839

Muñoz, N 71, 1388

Murakami, S 1234

Muramatsu, T 1522

Murphy, H 1578

Murray, GI 928

Murray, PG 586

Murray, T 354

Myklebost, O 1995

Naganuma, H 298

Nagao, K 25

Nagasawa, H 307

Nagata, C 1038

Nair, CKK 1584

Nakabeppu, Y 521

Nakagawa, K 1213

Nakagawa, S 1213

Nakagawachi, T 521

Nakagawara, A 1522

Nakajima, M 1735

Nakajima, Y 298

Nakakubo, Y 1234

Nakamura, M 1223

Nakamura, T 1101

Nakamura, Y 1522

Nakanishi, Y 726

Nakashima, T 1727

Nanni, O 47

Neal, DE 822, 1682

Negassa, A 263

Negoro, S 335

Negri, E 167

Nehls, O 217

Nelson, J 133

Neuhaus, P 733

Neuzil, J 161, 1948

Newton, R 1, 2002

Niethammer, D 1874

Nieweg, OE 175

Niitani, H 335

Nilsson, O 1747

Nishiguchi, S 1894

Nishikawa, T 1900

Nishiwaki, Y 808

Njor, SH 362

Noda, S 195

Noël, A 1111

Noguchi, M 195

Noguchi, S 1223

Noh, D-Y 910

Nomura, T 1584

Nooijen, WJ 175, 1346

Nord, C 36

Nörgråd, B 1687

Norman, AR 354, 1017, 1510, 1859

Norrback, K-F 593

Norrie, M 413

Northfelt, DW 1851

Nozawa, S 1883

Nozawa, Y 606

Nyari, TA 1370

Nyrén, O 1044

Nystrom, M 1516

Oates, J 1510, 1859

Obrist, P 574

O Byrne, KJ 1553

Occhino, M 1527

Oda, S 25

O Donnell, A 1128

O Driscoll, L 1077

O Dwyer, ST 1424

Offerhaus, J 1914

Ogawa, A 521

Ogawa, M 902

Oguma, Y 679

Oh, B-H 910

Ohashi, Y 335

Ohe, Y 808

O'Higgins, NJ 871, 1077

Ohmatsu, H 808

Ohmi, K 245

Ohnuma, K 455

Ohnuma, T 1038

Ohoka, Y 327

Ohsaki, Y 342

Ohshiba, T 1318

Ohtsu, A 11

Ohwada, S 1909

Okada, M 887

Okuno, S 951

Okushiba, S 1234

Old, LJ 937

Oliver, RTD 1516

Oliver, SE 1682

Olsen, AH 362

Olsen, J 1698

Olsen, JH 684, 832

Onda, T 245

O Neill, MA 1281

Onland-Moret, NC 1394

Oppelaar, H 283

O Reilly, S 895

O'Reilly, T 1622

Origasa, H 342

Orioli, P 1484

Ortega, E 940

Ortiz, AI 940

Ortner, M 217

Oshikiri, T 1234

Ostrowski, J 1143

Ota, I 327

Otten, W 1675

Oudet, P 1925

Overgaard, J 102

Owczarczak, B 1773

Owen, D 1756

Owens, PH 58

Pacquement, H 1925

Paffenbarger, RS 679

Paganelli, G 996

Pai-Panandiker, A 1327

Paisley, S 31

Palmer, MW 754

Palmieri, C 1971

Pang, D 373

Pani, G 1956

Paola, FD 320

Papamatheakis, J 1292

Papamichail, M 1292

Papapostolou, D 1111

Paparini, S 401

Parekh, R 579

Parhar, K 1756

Parikh, AA 796

Parker, L 1370

Parkinson, M 1077

Parry, JM 1271

Partin, JV 1615

Pasini, F 537, 1666

Passalidou, E 553

Pastorino, U 537

Patel, R 1851

Patel, S 579

Patel, V 599

Paterson, SC 983

Patnaik, A 1004

Paul, EA 1047

Pawlak, E 463

Paysant, J 1207

Pearce, A 1682

Pedersen, CG 658

Pedersen, KB 1995

Pedrazzani, C 1666

Peduto Eberl, L 788

Peeters, PHM 1394

Peiller, E 1794

Pellegriti, G 1537

Pelliccia, A 996

Pelosi, G 537, 1666

Penault-Llorca, F 1339, 1587

Perea-Milla, López, E 1702

Pérez, J 878

Perez, SA 1292

Périssel, B 516

Peros, G 206

Perotti, C 432

Perren, TJ 1578

Perret, GY 1987

Perricelli, A 1285

Persad, R 1682

Persing, DH 887

Peters, GJ 957, 1963

Peters, JPW 1439

Peters, TJ 1682

Peterse, JL 406, 707

Petersen, I 231

Petronzelli, F 996

Petry, K-U 1570

Pezzella, F 537, 553

Philip, I 181

Philip, R 613

Phillips, WA 270

Piccioli, F 1484

Piedbois, P 388

Pillai, G 553

Pillé, J-Y 1207

Pinedo, HM 957, 1963

Pinkerton, CR 1661

Piribauer, M 496

Pirotte, B 1111

Pistoia, V 1527

Planting, AST 814, 1199

Plebani, M 1239

Plummer, M 63, 1713

Płużańska, A 848

Pochet, L 1111

Pollán, M 96

Polyzos, A 1168

Pongracz, J 748

Ponthan, F 478

Porschen, R 217

Porter, DJ 1844

Porzio, G 1507

Posner, MR 11

Possinger, K 1406

Pratesi, G 965

Prior, Y 1017

Prise, KM 767

Probst-Hensch, NM 277

Pruschy, M 1786

Puget, S 516

Puisieux, A 1794

Purcell, R 1077

Purohit, A 630

Quaresima, B 1285

Raderer, M 1180

Raffaghello, L 1527

Rajkumar, T 1388

Ramazzotti, V 47

Ramp, U 918, 1801

Ranson, M 944

Rastkha, M 1721

Ratcliffe, P 1065

Ray-Coquard, I 181, 1721

Raynaud, F 1128

Razavi, D 502, 2004

Reboud-Ravaux, M 1111

Redondo, M 1702

Reed, MJ 630

Reed, SG 887

Rees, C 1128

Reeves, JR 1263

Reinke, F 648

Reitz, M 348

Rembiszewska, A 848

Remijnse, PL 406

Rémy, C 1439

Renardel de Lavalette, AC 447

Renehan, A 1424

Renier, A 388

Renner, C 937

Rettrup, B 424

Reuter, J 420

Rey, JA 109

Reyderman, L 1004

Reynaert, C 502, 2004

Reynolds, K 654

Rhodes, A 1017

Rhys-Evans, P 354

Ricchi, P 803

Riccobon, A 320

Ricevuto, E 1507

Richmond, I 1578

Ridder, MD 128

Ridolfi, R 320

Riedel, C 1248

Ries, C 1084

Riffaud, JC 1669

Rijken, PFJW 1439

Riley, RD 1191

Rinaldi, S 1394

Rindi, G 401

Ristimäki, A 1914

Ritter, G 937

Riva, A 965

Rizovski, B 782

Rizvi, SMA 944

Roberts, GT 202

Roberts, JT 843

Robinson, MC 1432

Robinson, SP 1592

Robson, CN 1432

Robson, W 822

Rocamora, A 940

Rocha, S 1786

Rochford, R 1566

Rochlitz, C 420

Rodella, S 47

Rodenhuis, S 406, 1091, 1831

Rofstad, EK 291

Rokana, S 1168

Rolhion, C 516

Romelsjö, A 1044

Roodenburg, JLN 1012

Roos, G 593, 1417

Rose, C 102

Rosenquist, R 593

Rosenthal, AN 251

Rosi, A 996

Ross, PJ 1157, 1859

Rossen, PB 658

Rossi, G 1851

Rosso, S 1702

Roth, AD 648

Roussel, J 1622

Rovere, GQD 354

Rowinsky, EK 1004

Roy, G 940

Royston, P 348

Rubenstein, J 1185

Rudd, R 1152

Ruevekamp, M 283

Ruhland, C 699

Ruiter, D 424

Rushbrook, SM 257

Rustin, GJS 1160

Rutgers, EJT 707

Ryan, A 251

Ryan, AJ 1592

Rzepka-Górska, I 848

Sabourin, J-C 863

Sacca, P 432

Sadaba, MC 940

Sagawa, N 606

Saijo, N 808

Saini, A 1859

Saito, M 1883

Sakamoto, I 1909

Sakamoto, M 726

Sakamoto, T 237

Sakarovitch, C 1239

Sakuma, S 1522

Salembier, GM 560

Salh, B 1756

Saller, R 1786

Salvatore, BA 1948

Samanta, T 1185

Sambiasi, D 491

Sanada, K 1543

Sánchez, J 96

Sanderson, C 567

Sanderson, M 1820

Sandstedt, B 478

Sanij, E 137

Santus, R 154

Sapino, A 702

Sasajima, Y 245

Sato, H 951

Sato, K 455

Sato, S 237

Sato, Y 726

Saunders, MP 1156

Sausville, EA 599

Sava, G 1484

Sawada, M 245

Scalliet, P 502, 2004

Schacht, V 1462

Schadendorf, D 1157

Scheffer, GL 878

Scheithauer, W 1180

Schellens, JHM 648, 814

Schenker, M 579

Scheper, RJ 878

Scherübl, H 115, 1217

Scherer, H 1217

Schernhammer, ES 79

Schittulli, F 491

Schlumberger, M 1537

Schlüns, K 231

Schmid, K 1180

Schmid, P 1406

Schmiegelow, K 775

Schmitt, M 918

Schneeweiss, B 1180

Schneider, A 1925

Schneider, J 104

Schoenberg, JB 50

Schöffski, P 648

Schofield, I 822

Schopp, B 1570

Schor, DSM 447

Schornagel, JH 1831

Schrama, JG 406, 1831

Schreiber, M 1277

Schrolnberger, C 782

Schüll, B 1180

Schull, WJ 382

Schulz, WA 1932

Schuppan, D 733, 1248

Schwartz, G 1004

Schwartz, W 362

Schweinhardt, P 478

Scollo, C 1537

Scorilas, A 1101

Scott, IS 257

Scott, J 895

Sebag-Montefiore, D 1352

Sebban, C 181

Sedlaczek, N 733

Segers, K 560

Seifert, H-H 1932

Sein, J 175, 1346

Sekiguchi, M 521

Sekine, I 808

Selby, P 666

Sengupta, PS 654

Seon, BK 1424

Serlupi-Crescenzi, O 630

Serrano, S 1702

Sesso, HD 679

Sethi, T 1809

Seufferlein, T 699

Sezer, O 1406

Sgambato, A 1956

Shah, K 1584

Shah, V 1271

Shamash, J 1516

Shanks, JH 654

Sharmila, A 1388

Sharp, L 928

Shattuck-Brandt, R 1445

Sheen, AJ 1119

Shen, DW 1327

Shen, H 1878

Sherlock, DJ 1119

Shiau, A-L 1492

Shibata, K 342

Shibata, T 726

Shieh, T-Y 366

Shimada, K 1223

Shimamura, M 307

Shimamura, T 726

Shimizu, H 1038

Shimizu, N 1038

Shinkai, T 808

Shinohara, T 1234

Shiosaka, S 327

Shono, Y 530

Shousha, S 1071

Shu, XO 1820

Shuto, T 1894

Siapati, KE 1641

Sigfusson, BF 933

Silvestris, N 491

Simmons, L 1128

Singh, A 630

Sinn, U 217

Sinnett, HD 1071

Sipponen, P 1239

Skretting, A 291

Skurzak, H 1143

Slade, MJ 1071

Slade, RJ 654

Slotman, BJ 447

Sly, WS 1065

Small, M 1844

Smith, JS 63, 1713

Smith, KD 983

Smith, PJ 1310, 1649

Smith, RE 1851

Smith, SK 237

Smith-Sörensen, B 1995

Snijders, PJF 1388

Soejima, H 521

Soengas, MS 1786

Sohda, S 951

Song, Y-S 910

Sonzogni, A 537

Soomro, I 1549

Sorahan, T 1035

Sørensen, FB 102

Sørensen, HT 684, 1687

Soria, C 1207, 1631

Soria, J 1207, 1631

Soubry, A 718

Souchon, R 828

Spanakis, NE 206

Spatz, A 1764

Speizer, FE 79

Spenger, C 478

Spijkervet, FKL 1012

Spizzo, G 574

Spooner, RA 1622

Springer, CJ 1622

Springer, ING 1105

Ssemwogerere, A 1708

Stagge, V 699

Stagnaro, E 47

Stanczyk, F 277

Stanton, PD 1263

Starzec, A 1987

Statkevich, P 1004

Stavroyianni, N 1168

Stead, ML 666

Stearns, ME 1605

Steele, JP 1516

Steers, G 553

Steger, GG 782

Stein, H 123, 1217

Steineck, G 84

Stelmachów, J 848

Stenning, SP 843

Stephan, C 1101

Stephenson, LP 66

Stern, PL 257

Steurer, M 574

Stevens, MFG 470, 599

Stewart, FA 283

Stewart, M 553

Steyerberg, EW 843

Stockton, D 74

Stoeltzing, O 796

Stokman, MA 1012

Stolz, B 1622

Storme, GA 120

Stoter, G 1199

Stovall, M 382

Straetmans, N 855

Strain, JJ 895

Stratford, M 1160

Stribbling, SM 1622

Su, AW 1327

Sugiura, T 335

Suhr, MAA 1105

Sumba, OP 1566

Sumerel, LA 1411

Sumpter, K 1017

Sun, J 760

Sundström, C 593

Sung, JJY 1560

Sung, L 1185

Supino, R 965

Suter, CM 413

Sutton, AJ 1191

Suzuoki, M 1234

Swiatkowski, S 1932

Swindell, R 654

Szarewski, A 42

Szeimies, R-M 1462

Szende, B 132

Szymańska, T 848

Sørensen, FB 110

Taal, BG 1095

Tabone, M-D 1925

Tagliaferri, P 1285, 1971

Tago, K 1909

Tait, D 1017, 1859

Takada, Y 327, 335

Takeda, A 796

Taketani, Y 1213

Takeuchi, H 1277

Takeyama, N 1038

Takeyoshi, I 1909

Takiguchi, Y 25

Takizawa, S 1213

Takizawa, T 740

Talianidis, I 1963

Tamba, M 951

Tamura, F 902

Tamura, T 808

Tanabe, N 25

Tanaka, H 530, 1894

Tanaka, S 902

Tange, UB 832

Tani, M 530

Tani, N 327

Tanimura, H 530

Tasaka, K 298

Tassone, P 1285, 1971

Tatsumi, K 25

Tayeb, MT 928

Taylor, I 163

Tchekmedyian, NS 1851

Tchirkov, A 516

Tebbutt, NC 1510

Teerlink, T 447

Teiten, M-H 154

Tejerina, A 104

Temam, S 1741

ten Hagen, TLM 314

Terheyden, H 1105

Terracciano, L 420

Terrett, JA 579

Terrier, P 1925

Terzi, A 537, 1666

Terzis, AJ 463

Tessier, JJ 1592

Thach, TQ 2006

Thierry, N 1111

Thievessen, I 1932

Thomas, JM 510

Thompson, AM 1281

Thompson, PI 1844

Thomsen, BL 382

Thomsen, LL 1135

Thorlacius, S 933

Thorpe, P 1071

Thrasher, AJ 1641

Thunberg, U 593

Tian, Z 944

Tiemann, M 1105

Timmermans, DRM 1675

Timorek, A 848

Tincello, DG 839

To, KF 1560

Tobin, G 593

Tofazzal, N 1271

Tolcher, AW 1004

Tollenaar, RAEM 1675

Tomek, S 167

Tomlinson, I 413

Tonetti, DA 1400

Tounekti, O 1764

Tozlu, S 863

Trapani, V 599

Trassard, M 1741

Travagli, JP 1537

Treilleux, I 1587

Trepel, JB 599

Trigo, J 1128

Trivella, M 553

Trott, P 354

Tryggvadottir, L 933

Tsai, C-C 366

Tsai, C-S 1492

Tsavaris, N 1168

Tse, PCH 1560

Tsujikawa, K 1223

Tsukazaki, K 1883

Tsunematsu, R 245

Tsunoda, T 530

Tubiana-Mathieu, N 1669

Tufail-Hanif, U 1809

Tumino, R 47

Turnbull, LW 4

Turner, A 1128

Twal, WO 871

Tyson, K 579

Uenishi, T 1894

Ueno, T 1229

Ugurel, S 1157

Ułańska, M 848

Ulrich-Pur, H 1180

Umemoto, M 237

Unger, E 1773

Ungersböck, K 496

Urbański, K 848

Uto, Y 307

Vaccarella, S 1388

Vago, P 516

Valabrega, G 396

Valduga, F 1666

Valle, JW 1156

van Asperen, CJ 1675

van Ballegooijen, M 1054

van Beest, P 718

van Beuningen, D 1470

Van Camp, B 855

van Capel, T 957

Van Dam, P 718

van de Kasteele, W 1346

van de Kasteele, WF 175

van den Akker, E 1570

van den Akker-van Marle, ME 1054

van den Bent, MJ 814, 1199

van den Berg, J 447

van den Berg, TK 447

Van den Berge, DL 128

van den Bergh, H 1781

Van den Brande, J 648

Van der Born, K 957

van der Burg, MEL 814, 1199

van der Gaast, A 814

Van der Kogel, AJ 1439

Van der Sanden, BPJ 1439

van der Wilt, CL 1963

van de Vijver, MJ 406

van Dijk, S 1675

Van Doornum, GJJ 1095

van Etten, B 314

van Herck, E 560

van IJken, MGA 314

van Leeuwen, FE 707

van Loenen-Frosch, F 1570

Van Marck, EA 718

van Noord, PAH 1394, 1818

Van Noorden, S 1071

van Oosterom, AT 1979

van Rees, B 1914

Van Riet, I 855

van Rijn, J 447

Van Roy, F 718

van Tiel, ST 314

van Veen, RN 1199

van Wering, ER 775

van Zandwijk, N 814

Vanbrabant, AS 560

Vanderkerken, K 855

Vanier-Viornery, A 1794

Vannier, J-P 1207, 1631

van t Veer, LJ 1091

Varis, A 1914

Vashisht, R 1071

Vasse, M 1207

Vassy, R 1987

Vaughan, L 1773

Veerman, AJP 775

Veerman, G 1963

Veitl, M 496

Vejborg, I 362

Veltman, SJ 1199

Venuta, S 1285, 1971

Verbeken, E 1979

Verdoliva, A 996

Vereecken, AJ 560

Vergouwe, Y 843

Verlato, G 1666

Vermeulen, PB 718

Vermorken, JB 648

Verovski, VN 128

Verra, N 1346

Verrelle, P 516

Verweij, J 814, 1199

Viale, G 537

Vidal, H 438

Vidaud, M 863

Viganò, C 47

Vilain, MO 1587

Villadsen, E 362

Villanacci, V 401

Villar, LM 940

Vincent, L 1207

Vincent-Salomon, A 1587

Vindigni, C 47

Vineis, P 47

Viscomi, C 1971

Vitali, P 320

Vivo, C 388

Voegeli, A-C 1925

Voelker, M 1248

Vonderhaar, BK 1301

von der Maase, H 658

von Lampe, B 1217

Voogd, AC 707

Vuong, V 1786

Vyth-Dreese, FA 175, 1346

Wachsmuth, R 1920

Waddell, K 2002

Wagenaar-Miller, RA 1445

Wagnières, G 1781

Wakai, K 1522

Wakeman, JA 202

Waller, DA 1553

Waller, J 42

Waller, S 1160

Wanders, J 648

Wandert, T 348

Wang, B 1878

Wang, H 951

Wang, M 1605

Wang, T 887

Wang, W 624

Wang, W-M 1866

Wang, W-Y 187

Wang, WC 1773

Wani, KMY 1584

Ward, RL 413

Wardle, J 42

Wasson, GR 895

Watanabe, R 25

Watanabe, Y 887

Waters, CM 1809

Waters, JS 354

Waterton, JC 1592

Watson, PH 1065

Wauters, N 120

Weigang-Köhler, K 648

Weigelt, B 1091

Wein, A 1248

Weir, DG 895

Welch, RS 654

Welt, S 937

Wenzel, C 496, 782

Wenzel, M 918

Wernecke, KD 1406

Weterman, M 1914

Wheatley, DN 613

Whiteside, TL 223, 1301

Wildiers, H 1979

Wilkinson, N 1578

Wilkinson, PM 654

Willett, WC 1381

Wilson, DJ 125

Wilson, G 654

Wilson, GE 257

Wilson, P 1516

Wilson, TJ 137

Wiltshire, M 1310

Winther, JF 382

Wise, JP 58

Wit R, de 1823

Wocial, T 1143

Wolf, D 574

Wolfbeis, OS 1462

Wong, C-M 2006

Wong, J 1185

Wong, J-M 1866

Wood, J 1622

Wooster, R 510

Wotherspoon, A 354, 1017

Wright, EG 548

Wu, C-L 1492

Wu, C-W 1058

Wykoff, CC 1065

Xu, D 145

Yakushiji, H 521

Yamada, T 245, 1909

Yamamoto, H 1223

Yamamoto, T 11, 1894

Yamamoto, Y 307

Yamashita, H 1223

Yamaue, H 530

Yamochi, T 455, 455

Yanaihara, A 237

Yane, K 1223

Yano, T 1213

Yasugi, T 1213

Ye, W 1044

Yeh, GC 599

Yen, M-F 1866

Yip, PSF 2006

Yokomise, H 1727

Yokoyama, Y 237

Yongwei, Y 231

Yoshikawa, H 298, 951, 1213

Yoshimura, K 808

Yoshimura, S 1909

Young, L 586

Young, NL 1185

Yousef, GM 1101

Yu, H 263

Yu, J 1560

Zachariae, R 658

Zahm, SH 58

Zanetti, R 1702

Zanon, E 702

Zappa, M 1175

Zarrilli, R 803

Zekri, JM 1335

Zeng, H 760

Zhang, B 58

Zhang, F 1878

Zhang, H 1375

Zhang, Y 58

Zheng, T 58

Zheng, W 1820

Ziche, M 1484

Zieliński, J 848

Ziółkowska, I 848

Zoeteweij, MW 1675

Zoula, S 1439

Zucchetti, M 965

Zunino, F 965

Zurita, AJ 878

